# Supplementary figures and images for: CXXC5 Mediates DHT-Induced Androgenetic Alopecia via PGD2
Source: Cells. 2023 Feb 9;12(4):555. doi: 10.3390/cells12040555 (PMC9954685; doi:10.3390/cells12040555)

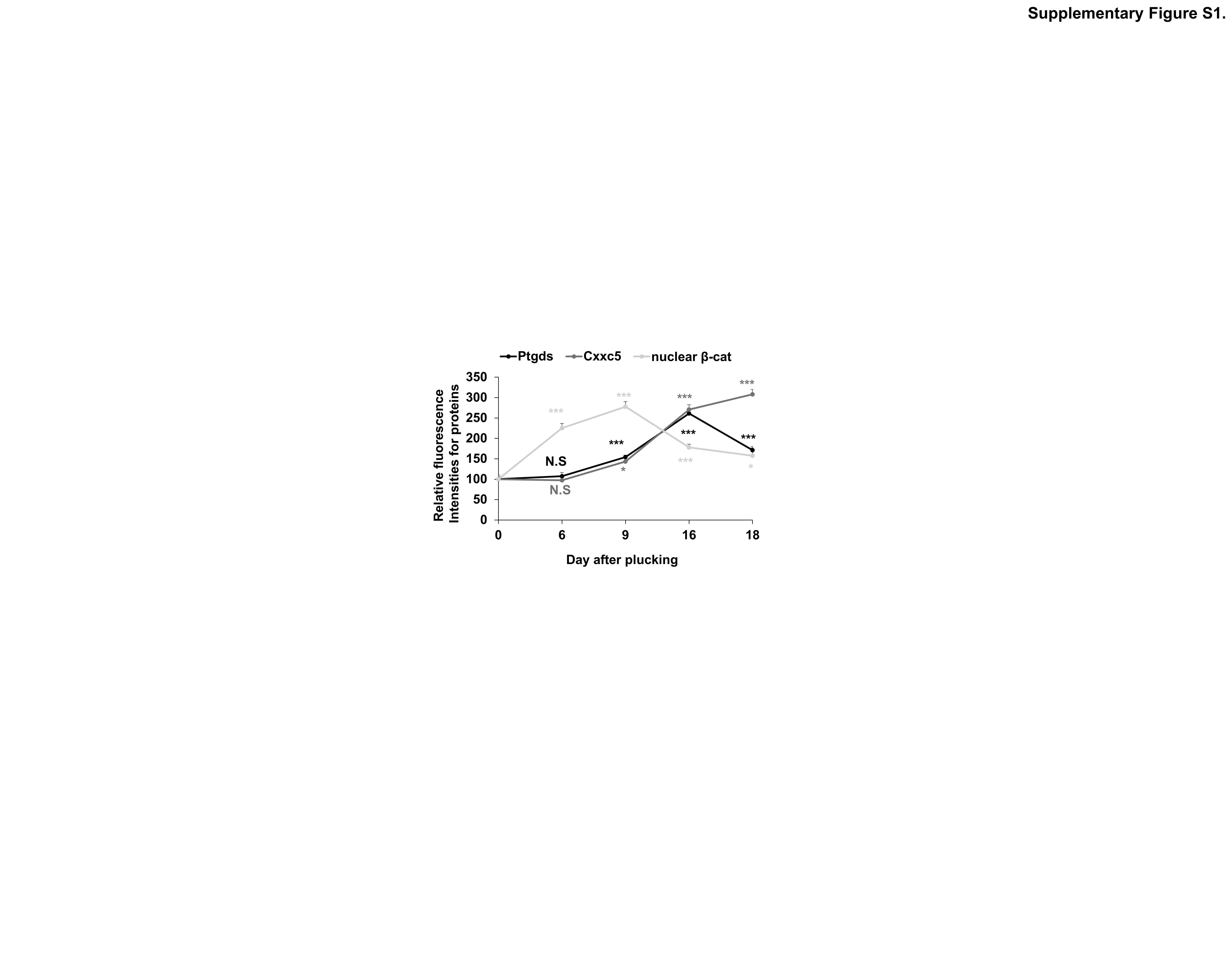

Supplement: Supplementary file 1 [file cells-12-00555-s001.zip › Supplementary Figure S1.TIF]

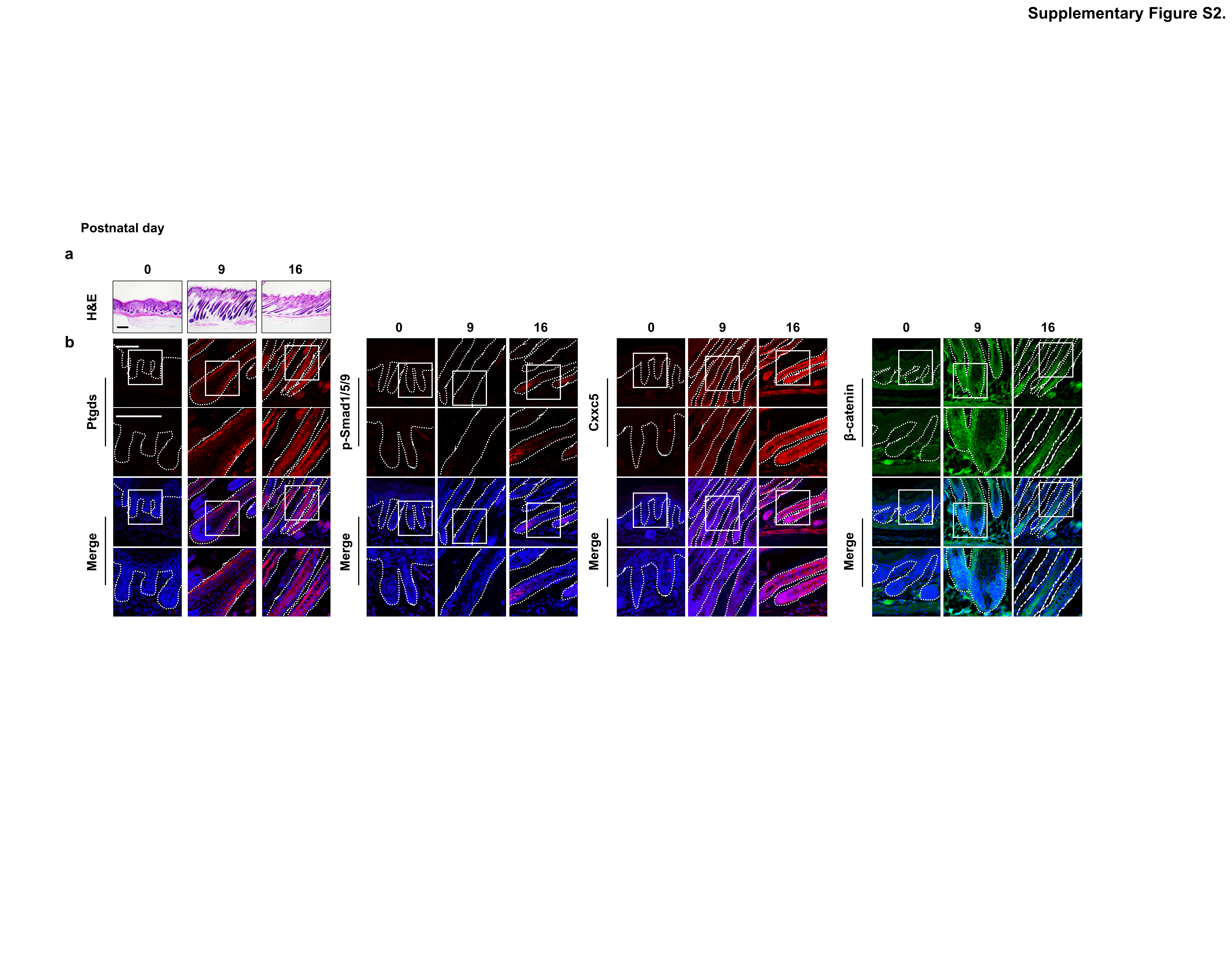

Supplement: Supplementary file 1 [file cells-12-00555-s001.zip › Supplementary Figure S2.TIF]

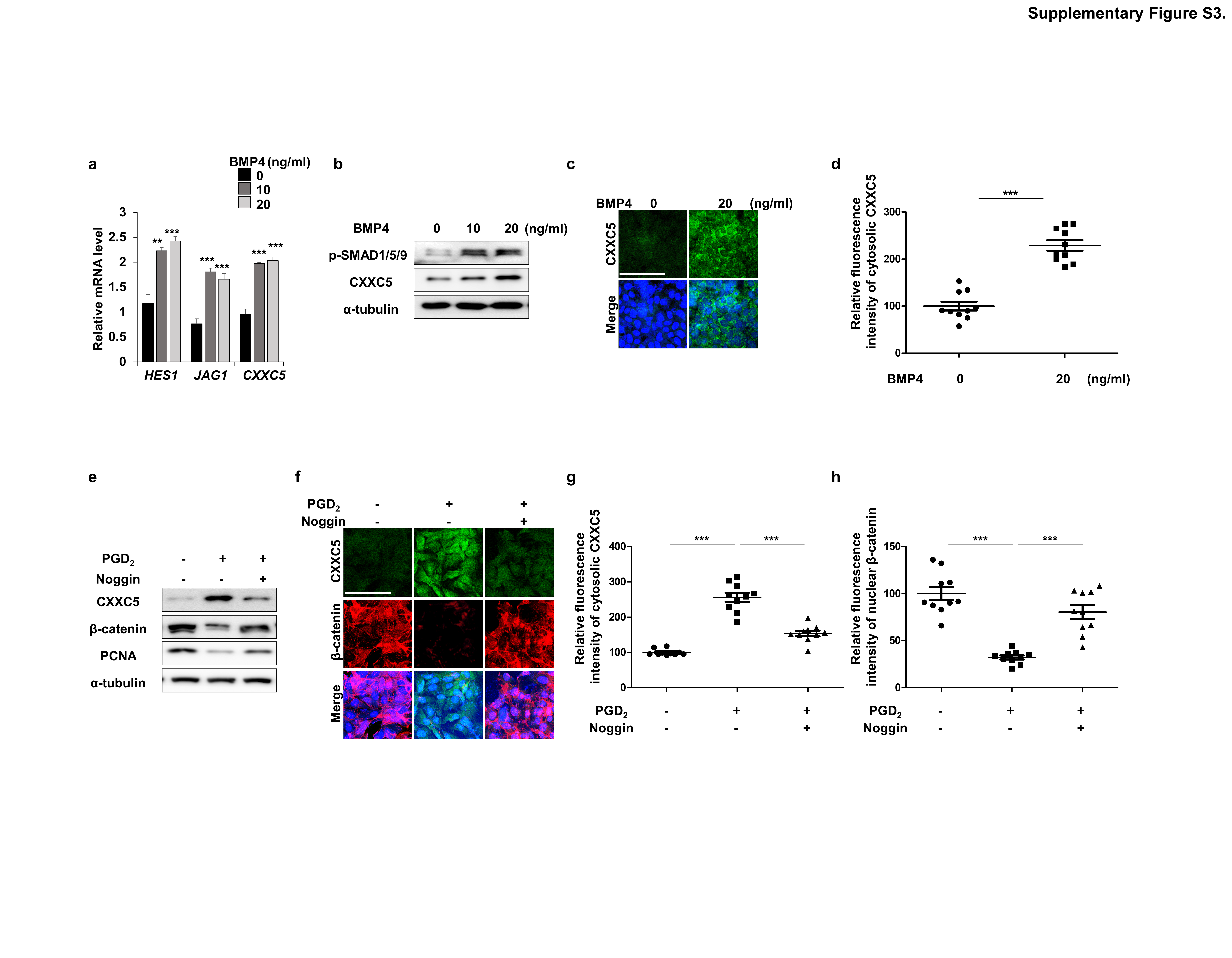

Supplement: Supplementary file 1 [file cells-12-00555-s001.zip › Supplementary Figure S3.TIF]

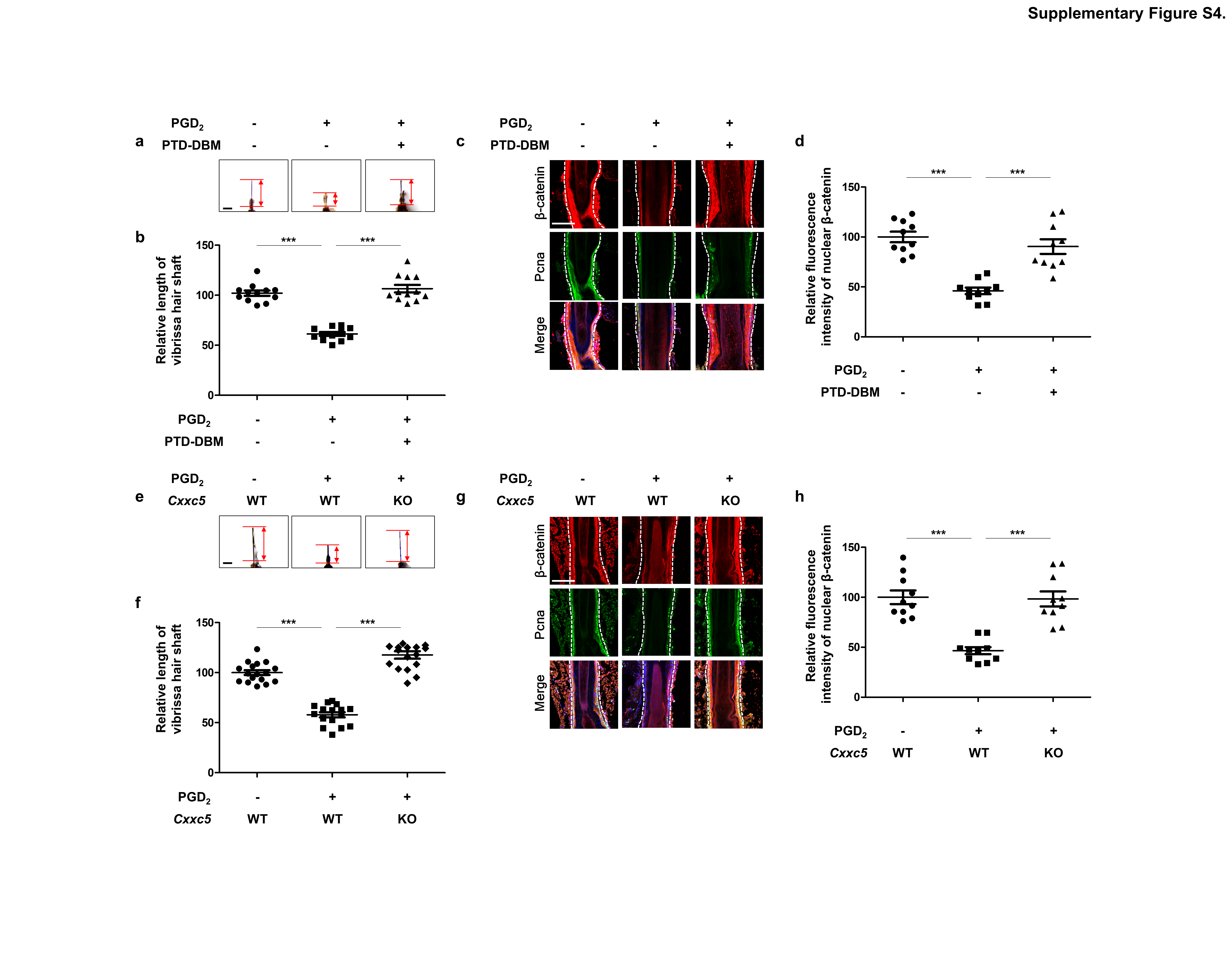

Supplement: Supplementary file 1 [file cells-12-00555-s001.zip › Supplementary Figure S4.TIF]

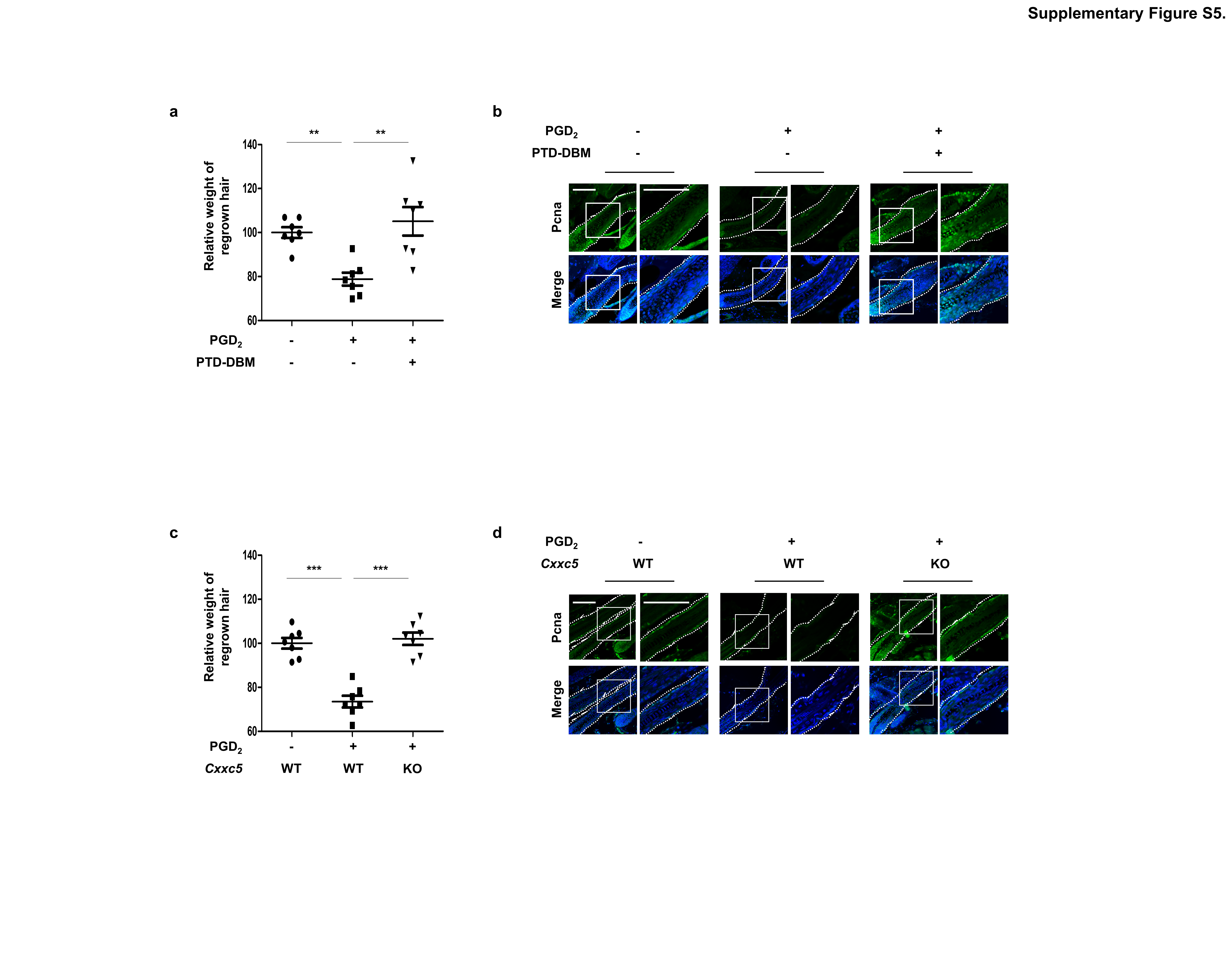

Supplement: Supplementary file 1 [file cells-12-00555-s001.zip › Supplementary Figure S5.TIF]

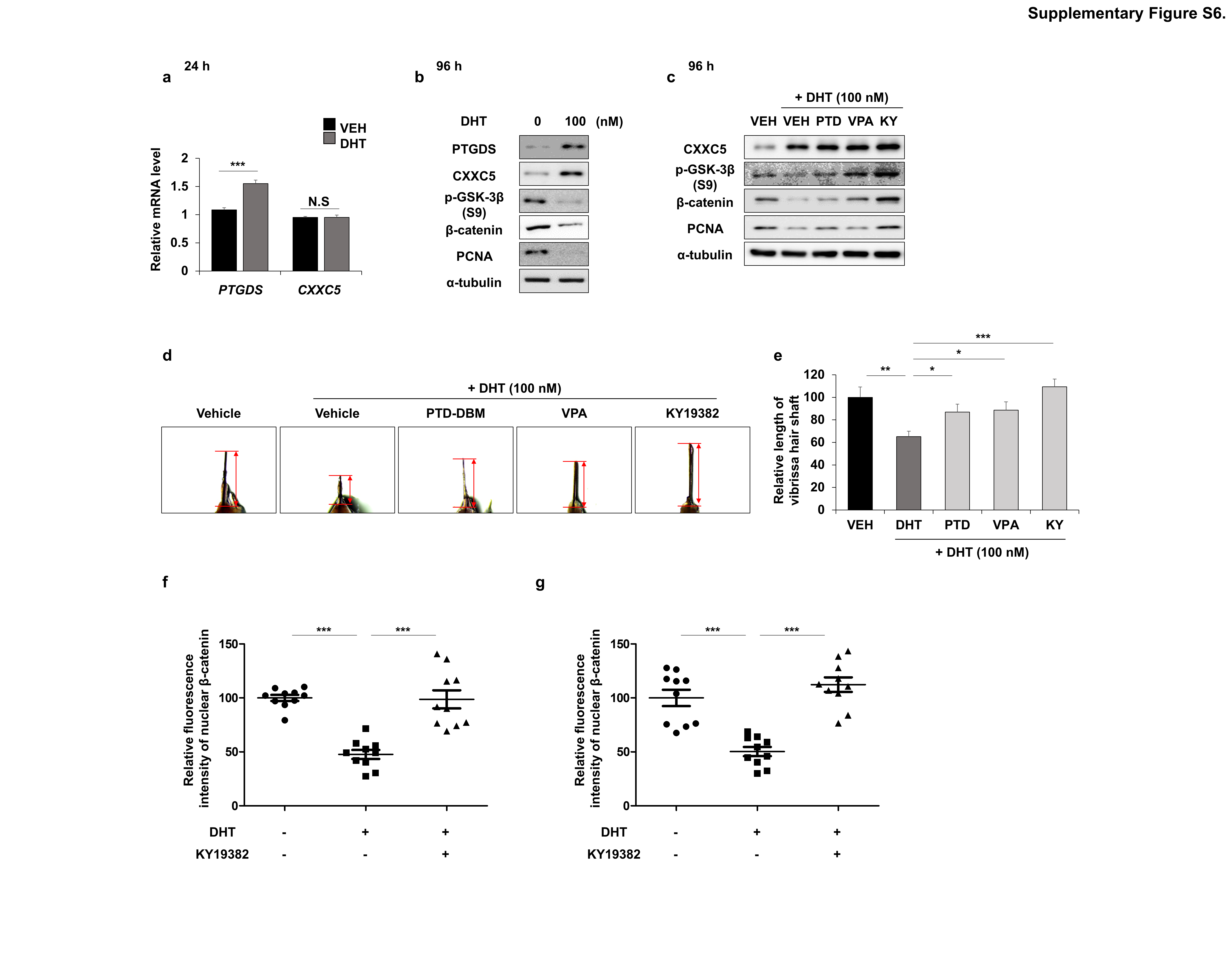

Supplement: Supplementary file 1 [file cells-12-00555-s001.zip › Supplementary Figure S6.TIF]

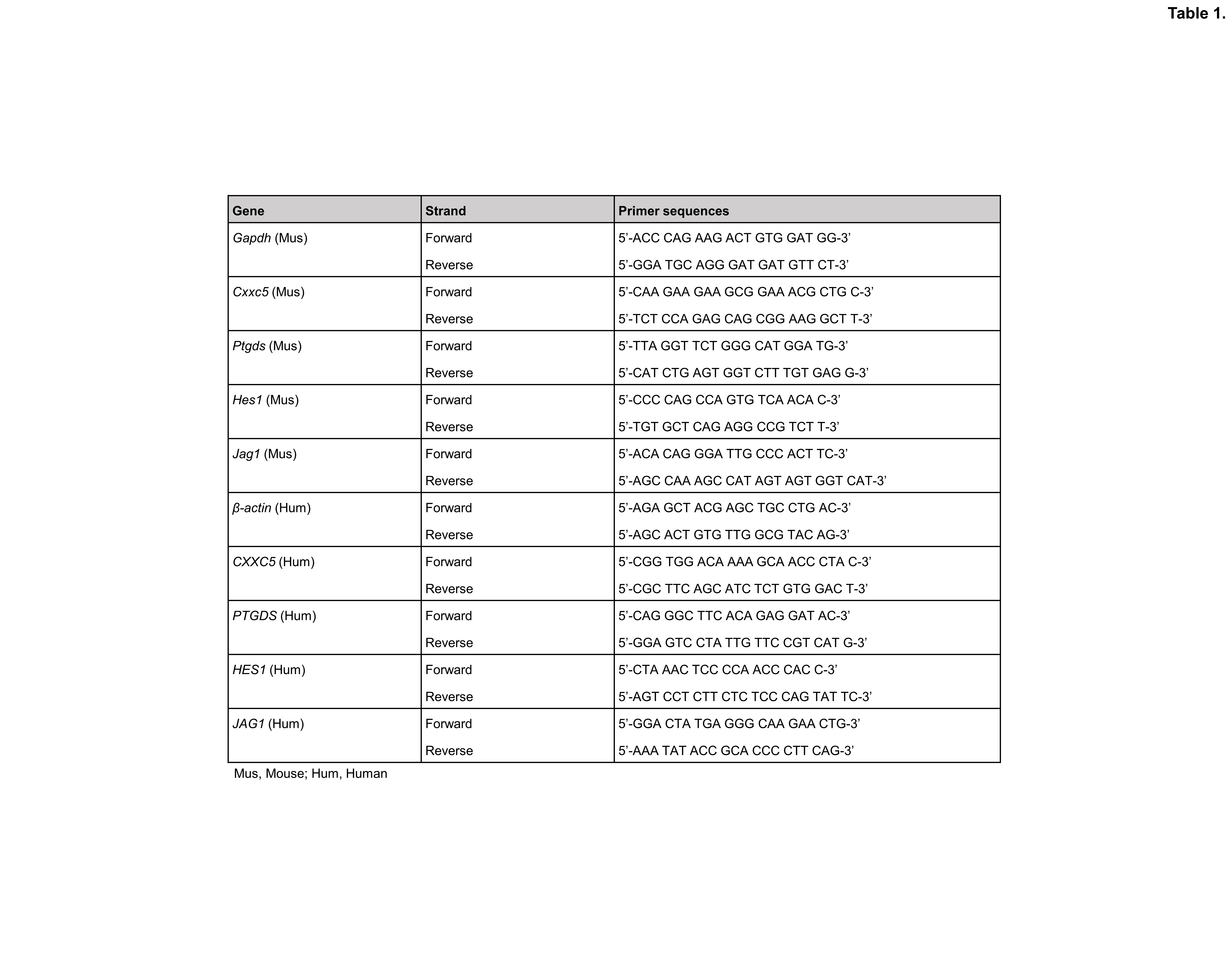

Supplement: Supplementary file 1 [file cells-12-00555-s001.zip › Table S1.TIF]
